# Global weirding at mass extinction horizons

**DOI:** 10.1093/nsr/nwad206

**Published:** 2023-07-25

**Authors:** Gregory J Retallack

**Affiliations:** Department of Earth Sciences, University of Oregon, USA

A strong motivation for studying mass extinctions is understanding how current climatic and biodiversity crises will affect creatures living on land such as ourselves. Perhaps the question is: how bad can it get? In that case, terrestrial Permian-Triassic mass extinctions are an irresistible worst-case scenario [1]. But the devil is in the details of finding well-dated records of complex, short-term environmental fluctuations. There are many unexpected events associated with mass extinctions, not only soaring CO_2_ and temperatures, but fires, droughts, floods and soil erosion, as we are finding currently, with changes best described as ‘global weirding’ [2].

**Figure 1.**
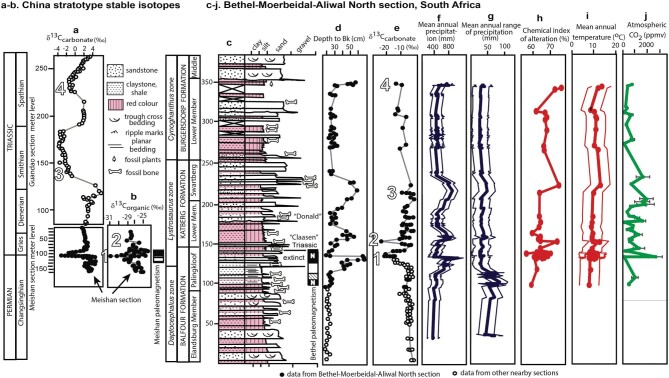
Late Permian to Middle Triassic isotopic and paleosol perturbations near (a, b) Meishan China and (c–j) Bethulie, South Africa. Isotopic perturbations from atmospheric pollution by large basaltic eruptions of the Siberian traps allow linking of marine and non-marine sections [3]. Depth to Bk in paleosols is the basis for mean annual precipitation estimates, and thickness of Bk is the basis for mean annual range of precipitation [8]. Chemical index of alteration and paleotemperature is from a major element chemical composition of the paleosols [1], and atmospheric CO_2_ from stable isotopic composition of paleosols [12]. Isotopic and paleosol Bk data from Bethel farm (solid symbols) are supplemented with data from other nearby South African sections (open symbols) [1]. The image is reprinted with permission from CCC 1363214–1.

Deep-time records of mass extinctions may be dismissed as of no relevance to current crises because of their poor temporal resolution, however some records of the Late Permian crisis are in varved shales with fossilized autumn leaves as indications that they are annual. Within these varved shales major carbon isotopic excursions that correlate with mass extinctions are within centuries [3]. Sequences of paleosols across the Permian–Triassic transition are generally weakly developed, representing only centuries or a few millennia of soil formation [4], and that is their effective temporal resolution. Although this brief perspective uses the Karoo Basin of South Africa as an example (Fig. 1), comparable greenhouse spikes, climatic volatility, stable isotopic anomalies, and successive extinction pulses are now well known from multiple sites in Australia, Antarctica and Russia [3–5].

Although marine shales have been regarded as high-resolution records of past events, they do not rival the terrestrial record across the Permian–Triassic boundary. The great mass extinction was by definition Late Permian, because it is 17 cm below the Permian–Triassic boundary defined by conodonts in the stratotype section of Meishan China [6]. Strictly speaking then, the great extinction is not ‘end-Permian’ or even ‘latest-Permian’, but Late Permian or Changsinghian. That 17 cm is represented by 12 m in South African sections (Fig. 1), and other Gondwanan terrestrial sections also are many meters thick, orders of magnitude thicker then Meishan [5,7]. This allows clear resolution of dramatic swings in precipitation, seasonality and paleotemperature on land from standard proxies derived from paleosol physical and geochemical data. Mean annual precipitation (Fig. 1f) and mean annual range of precipitation (Fig. 1g; difference between driest and wettest month) can be estimated from the depth and thickness of paleosol calcic horizons, by comparison with modern soils [8]. Overall chemical weathering (Fig. 1h) and mean annual temperature (Fig. 1i) can be estimated from chemical composition of paleosols [1]. These results confirm that rising temperatures coincide with rising precipitation and chemical weathering, but not necessarily with seasonality of precipitation.

Although the whole Early Triassic has been regarded as a protracted time of persistent environmental misery [9], that conclusion is an artefact of binning data into long time intervals. The record preferred here has meter level resolution (Fig. 1), and shows multiple hits and recovery from disaster. A case has been made for drought as a killer, with spectacular cases of fossil mummies of *Lystrosaurus* [10], but these stratigraphic levels with evidence of drought are separate from the extinction horizons [11], all characterized by high warmth and precipitation [Fig. 1]. These differences by themselves are not killers; rather the displacement of O_2_ by CO_2_ killed plants at the root by soil air anoxia, and animals by a variety of symptoms like high altitude pulmonary edema [3].

Atmospheric CO_2_ remains the best explanation for the greatest, as well as other, mass extinction levels of the Permian and Triassic, with emissions largely from several gigantic eruptions of the Siberian Traps creating thermogenic CH_4_ and CO_2_ from intrusion of coals [3]. Ancient CO_2_ levels can be inferred from both the stable isotopic composition of paleosol carbonate and from the stomatal index of fossil leaves [11]. Discovery of a herbarium leaf of *Ginkgo* picked in 1754, and expanded analyses of such leaves now, allows modification of the proxy formula for calculating CO_2_ from stomatal index [12]. The new proxy also yields results close to those derived from the calcareous paleosol carbon isotopic method [12]. So, the ‘How bad can it get?’ question can now be answered, with a new estimate of ∼2126 ppm for atmospheric CO_2_ at the greatest mass extinction [12]—much lower than past estimates. We are currently at 424 ppm, up considerably from a full glacial 180 ppm, so only about a ninth of the way to disaster, but the lesson from the past is that fluctuations and unpredictability can be expected.
